# ESTES recommendations regarding distal radius fractures

**DOI:** 10.1007/s00068-026-03203-7

**Published:** 2026-05-11

**Authors:** C. M. Lameijer, M. P. J. Teuben, E. A. K. van Delft, M. Tomazevic, M. Kastelec, Ch. Nau

**Affiliations:** 1 Department of Traumasurgery, Amsterdam University Medical Center, Amsterdam, The Netherlands; 2 Department of Traumatology, University Hospital Zurich, Zurich, Switzerland; 3 Department of Orthopedic Traumasurgery, Faculty of Medicine, University Medical Centre Ljubljana, Ljubljana, Slovenia; 4 Department of Traumatology, Orthopedic Surgery and Handsurgery, Klinikum Worms, Worms, Germany

**Keywords:** Distal radius fracture, Recommendations, Treatment, Rehabilitation, Elderly

## Abstract

Distal radius fractures (DRFs) account for 15% of all fractures in adults. Optimal treatment of DRFs depend on patient and fracture characteristics. Non- or minimally displaced DRFs can be treated nonoperatively with a cast. Although surgical treatment is becoming more popular, the majority of patients with a DRF are still treated nonoperatively. In addition, these considerations may be different for elderly patients. Conflicting evidence exists on the different aspects of treatment of DRFs. These recommendations regarding distal radius fractures describe considerations regarding diagnostic modalities, associated soft tissue injuries, different treatment options, rehabilitation protocol, considerations for treatment of elderly patients, follow-up decisions and possible complications. The aim is to aid the orthopedic traumasurgeon in decision making and treatment of distal radius fractures.

## Introduction

Distal radius fractures (DRFs) account for 15% of all fractures in adults with annual incidences reported of 9/10,000 men and 37/10,000 women in patients aged 35 years and older [1–3]. DRFs have a bimodal division in incidence, with peak incidences in young (predominantly male) and older (predominantly female) patients [4, 5].

**Fig. 1:**
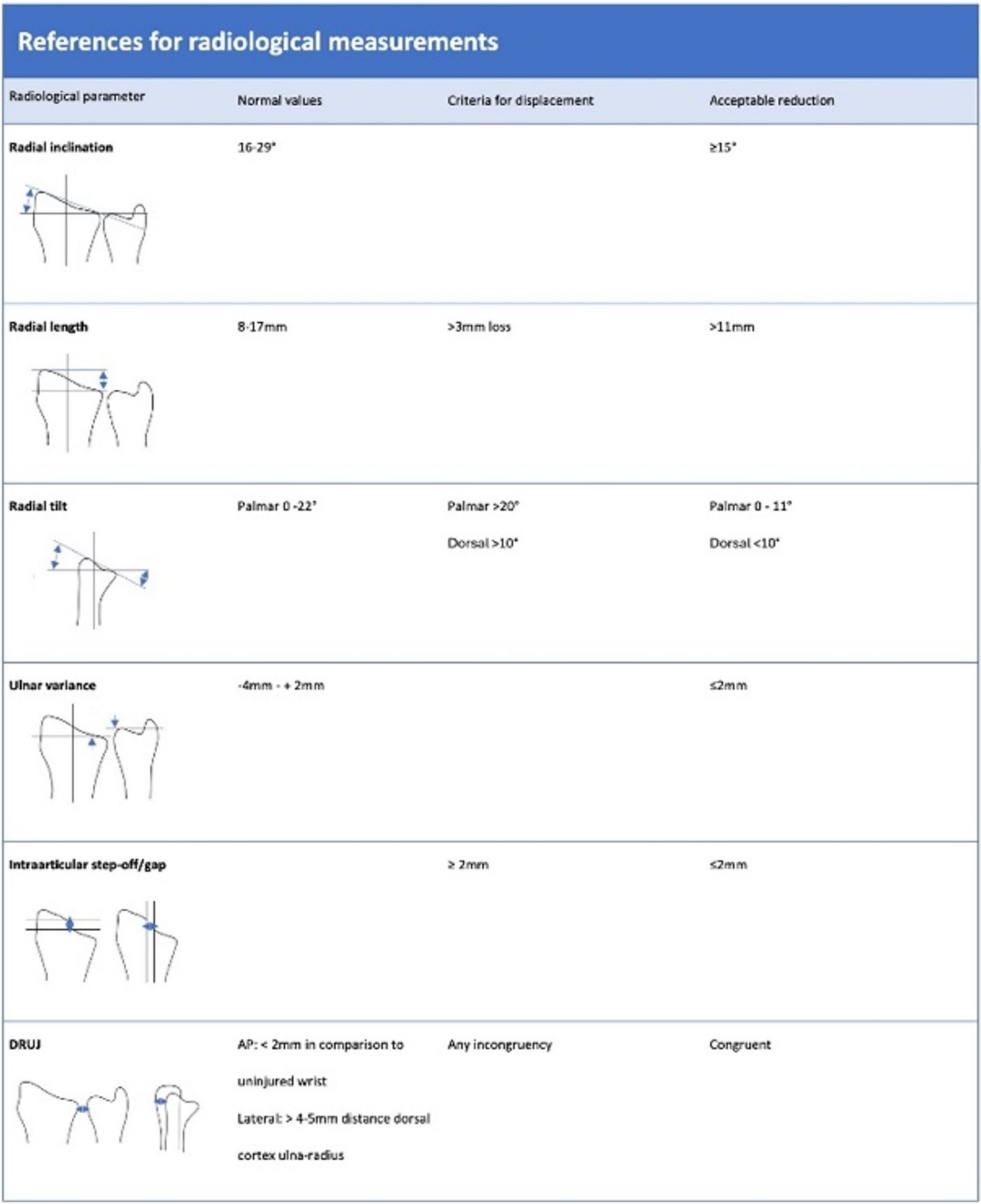
Radiological values regarding distal radius fractures [26–31]

A fall on the outstretched hand is the most common trauma mechanism. Fractures are classified as extra- or intraarticular and several classification systems exists, of which the AO/ASIF classification is widely used, see Fig. 1 [6]. Optimal treatment of DRFs depend on patient and fracture characteristics. Non- or minimally displaced DRFs can be treated nonoperatively with a cast. A large proportion of displaced DRFs can be treated successfully nonoperatively if acceptable reduction is achieved and secondary displacement does not occur in the follow-up. Otherwise, when a nonacceptable reduction is present, surgical treatment of DRFs is advocated. Surgical treatment strategy mainly includes volar plating, while dorsal plating can be necessary if dorsal comminution is present [7, 8].

Although surgical treatment is becoming more popular, the majority of patients with a DRF are still treated nonoperatively [9–13]. Conflicting evidence exists on the different aspects of treatment of DRFs [14–17]. The Cochrane Collaboration is unable to recommend on nonoperative or surgical treatment, reduction techniques, casting duration and rehabilitation [14, 18]. Also, the American Academy of Orthopedic Surgeons (AAOS) did not publish a clear recommendation [19].

Outcomes following DRFs can be depicted using three different modalities; clinician reported outcomes (CROs) measuring range of motion and grip strengthand patient reported outcomes (PROs) using validated questionnaires to capture subjective outcome as perceived by patients. In addition, radiological outcome can be captured with restoration of alignment, articular congruency, malunion, nonunion or posttraumatic arthritis. The impact of the treatment of choice for distal fractures should be guided by clinical relevance.

These recommendations regarding DRFs will describe considerations regarding diagnostic modalities, associated soft tissue injuries, different treatment options, rehabilitation protocol, follow-up decisions and possible complications. The aim is to aid the orthopedic traumasurgeon in decision making and planning of DRFs.

## Diagnostics and imaging

The diagnostic evaluation begins with medical history, followed by a clinical examination, and is completed with appropriate imaging. Key factors include the patient’s age, pre-injury activity level, and functional demands for future wrist use. Comorbidities and ongoing medications also significantly influence treatment decisions [20, 21].

Regarding the fracture, it is essential to determine the mechanism of injury and energy involved. A fracture resulting from a fall from standing height is approached differently from one sustained in a high-energy event such as a motor vehicle accident or a fall during high-speed sports activity [22].

On physical examination, swelling and skin color should be documented. A visible wrist deformity may be present in both the anteroposterior and medio-lateral planes [23]. Neurovascular examination includes motor testing of the median, radial, and ulnar nerves, sensory evaluation of their respective territories, and assessment of capillary refill in the fingers. Due to swelling, the radial pulse may be difficult to palpate, if in doubt, use ultrasound to confirm pulses is useful [24]. The painful area is palpated gently, avoiding unnecessary aggravation of symptoms [25].

Range of motion in adjacent joints should be assessed before evaluating the radiocarpal joint itself. Evaluation of rotational movements of the forearm is particularly important [23].

Standard radiographic evaluation is performed in anteroposterior (AP) and lateral projections. When a scaphoid fracture is suspected, two radiographs with oblique navicular projections are performed or additional CT imaging should be considered.

Radiographic assessment requires the recognition and measurement of specific parameters (Fig. 1) [26–31]. Fracture displacement should be identified according to the radiographic parameters visualized in Fig. 1. In fractures with marked displacement, these parameters should be reassessed after reduction.

Computed tomography (CT) is indicated when standard radiographs do not allow accurate assessment of these parameters, or when there is suspicion of significant articular displacement. CT imaging is particularly useful for preoperative planning in complex fractures where conventional X-rays are insufficient for defining the fracture pattern [32]. CT is far more consistent than radiographic imaging, because the latter shows large variability between and within observers. Therefore, CT is preferred when precise and reliable measurements are required or when recognition of fracture patterns is necessary for planning surgery [33]. MR imaging does show soft tissue injuries or occult fractures, but it is not used in an acute evaluation of DRFs [34].

## Classification

Due to the high frequency of distal radius fractures and the long-standing history of both non-operative and operative treatments, several classification systems have been developed. The most often used are the AO/ASIF, Fernandez classification or the four corner concept [22, 35–37].

### AO/ASIF classification

**Fig. 2:**
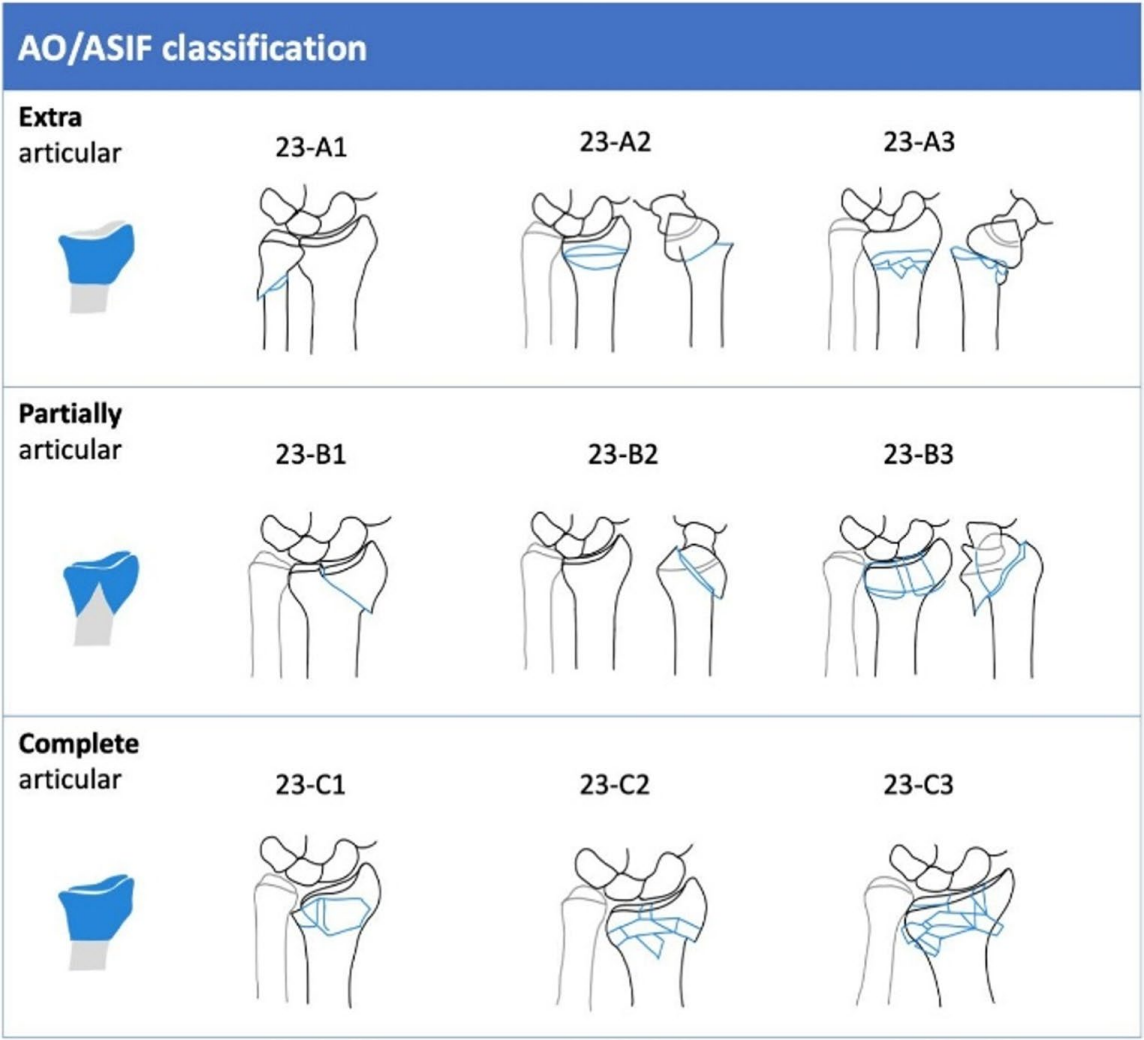
Distal radius fracture classification according to the AO/ASIF [6]

The AO/ASIF classification is the most anatomically detailed and regularly updated. It categorizes fractures by anatomical location (Fig. 2). Based on joint surface involvement, fractures are classified into; type A: extra-articular fractures, type B: partial articular fractures and type C: complete articular fractures with no continuity between the joint and diaphysis (Fig. 2).

### Fernandez classification

The Fernandez classification focuses on the mechanism of injury and displacement direction, providing insights into fracture stability and surgical management [37]. It emphasizes the fracture location, joint involvement, direction of displacement, stability, soft tissue injuries, and their impact on treatment.

**Fig. 3:**
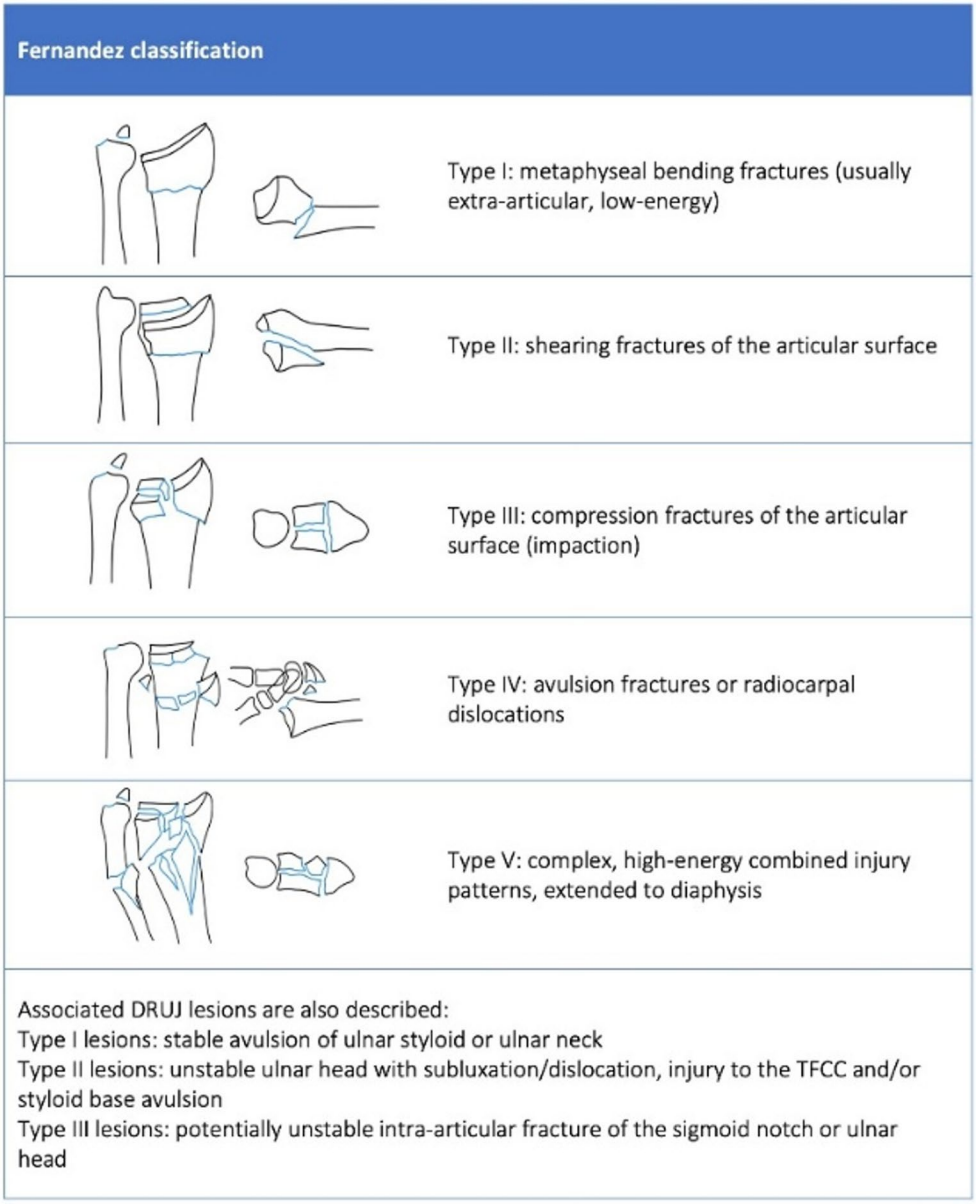
Distal radius fracture classification according to the Fernandez classification [22]

The classification consists of five main fracture types of the distal radius, which are the most important for guidance toward the treatment of distal radius fractures (Fig. 3) [22]. In addition associated DRUJ lesions are also described.

### The four corner concept

**Fig. 4:**
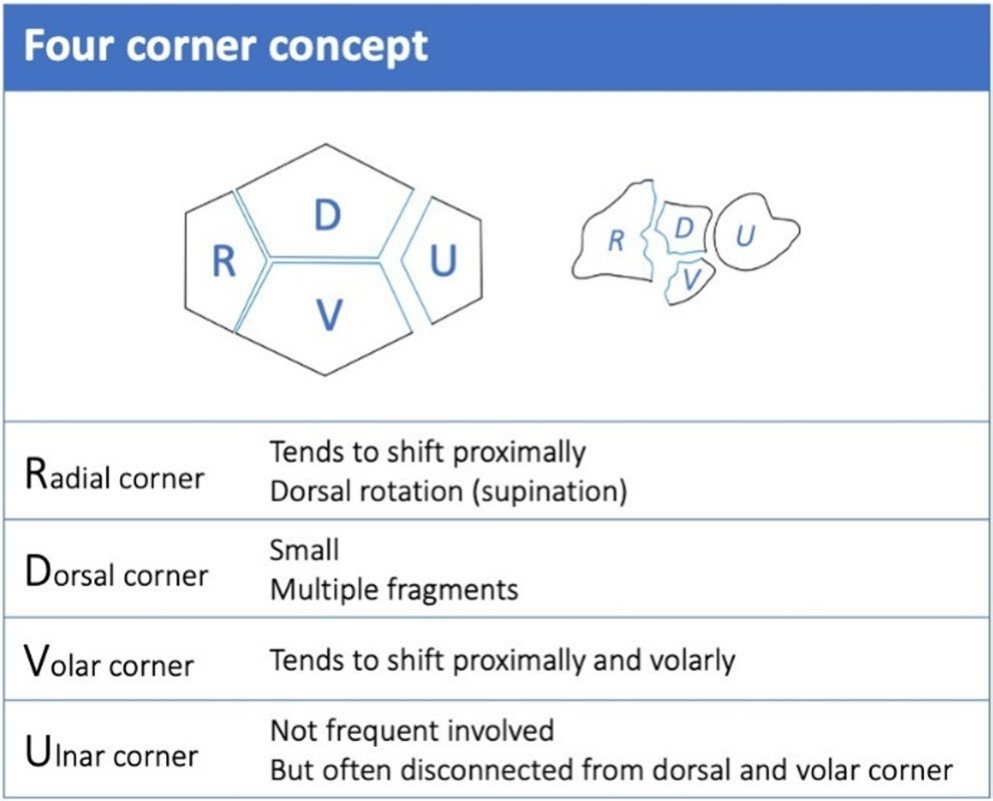
Four-corner concept for classifying distal radius fractures [38]

The four corner concept describes the distal radius and ulna divided into four biomechanical units (“corners”), each with unique roles in stability, mobility, and force transmission (Fig. 4). This four-corner concepts allows the identification of at least eight fracture patterns, ranging from extra-articular to complete intra-articular injuries. A key idea is the “key corner”, the fragment linked to the lunate bone, whose reduction and fixation are essential to restore joint congruency and prevent chronic subluxation. The radial corner is crucial for radiocarpal stability, the ulnar corner for distal radioulnar joint stability, while the dorsal and volar corners affect joint congruency and carpal alignment. This concept provides a framework for tailored surgical strategies to achieve anatomical restoration and optimal function after distal radius fractures (Fig. 4) [38].

In summary, the AO/ASIF classification offers detailed anatomical descriptions, while the Fernandez system and the four corner concept provide a biomechanical and treatment-oriented approach. Understanding these systems is crucial for accurate diagnosis, surgical planning, and communication among clinicians.

## Non-operative treatment

The overall incidence of non-displaced DRFs is estimated to be 30–40% [39]. This incidence varies among age groups. In younger patients (< 50 years of age), approximately 50% of the DRFs are non- or minimally displaced. In elderly, displacement occurs twice as often as non- or minimally displacement of DRFs [40]. Although operative treatment became more popular over the last years, the majority of patients with DRFs are still treated nonoperatively, with excellent functional outcomes [41–49]. Moreover, nonoperative treatment of DRFs has a lower complication rate compared with operative treatment with plate-osteosynthesis, 0–14 versus 17% respectively [50, 51].

The optimal treatment of DRFs depends on both patient-related and fracture-specific characteristics. Non- or minimally displaced DRFs can be treated nonoperatively with a cast. Also, a substantial proportion of displaced DRFs can be successfully treated nonoperative as long as the reduction is acceptable and secondary dislocation does not occur during follow up [50, 52, 53].

Once a fracture is displaced, reduction should be considered (Fig. 1). Closed reduction of displaced DRFs is usually performed at the emergency department with the intention to restore fracture alignment.

Techniques to perform a closed reduction at the emergency department are manual traction or finger-trap traction [54]. For closed reduction, some form of anaesthesia is required. Reported methods of anaesthesia are hematoma blocks, intravenous regional anaesthesia, regional nerve blocks and procedural sedation, without sufficient evidence of one method over another [55].

Criteria for acceptable reduction are presented in Fig. 1. In young patients however, optimal anatomic reduction is preferred, and risk factors for secondary displacement should be taken into account. Thirty percentof all primarily reduced fractures show secondary displacement. Risk factors that are associated with secondary displacement are dorsal comminution, ulnar variance and older age [56–58]. Therefore it is recommended to repeat radiographs 7–14 days after reduction. In case of secondary displacement that meet the criteria visualised in Fig. 1, surgical treatment is recommended. For elderly patients, considerations regarding treatment of secondary displacement may be different. These are described below in section “Considerations in the elderly patient” below. Repeated radiographs after a prolonged period of time do not seem to be beneficial, only in case of a clinical indication [59].

The nonoperative treatment of non- or minimally displaced DRFs or displaced and adequately reduced DRFs consists of cast immobilisation. Studies have shown no benefit of above-elbow casts compared to short arm casts in terms of secondary displacement, functional outcome or patient reported outcome measures [60–62]. Also, recent research shows no significant difference regarding secondary displacement or patient reported outcomes between the application of circumferential casting versus a plaster splint [63]. Although casts can be mouled in various positions after reduction, current evidence shows no clear superiority of one position of immobilisation over another in terms of functional, radiological or patient reported outcome [15, 64].

The desirable conservative treatment of DRFs is short, safe, comfortable for the patient and facilitates fast recovery, independency and early return to work or daily activities. In the past, non- or minimally displaced DRFs were immobilised for four to six weeks [65, 66]. Research indicated that a shorter period of immobilisation might accelerate and support functional outcome and does not compromise safety [67–69]. Recently studies have been published that prove that a shorter period of immobilisation is safe and might even lead to improvement of outcome. In a randomized trial on non- or minimally displaced DRF, three weeks of cast immobilisation yielded significant better functional outcome than five weeks of immobilisation.However, the difference did not reach clinical relevance [70]. This study group also analysed the period of cast immobilisation displaced and reduced DRFs. Four weeks of cast immobilisation showed comparable outcomes to six weeks, with a significant, although not clinically important, improvement in functional outcome and no adverse events [71]. The potential effectiveness of braces in the treatment of DRFs has been investigated for more than 25 years [72, 73]. This treatment has promising advantages according to cost effectiveness and patient satisfaction. Future studies will provide scientific evidence on this subject [74].

## Surgical treatment

Indications for operative treatment are shown in Table 1 [75]. Consensus is lacking regarding surgical interventions for closed DRFs. The timing of surgery depends upon the associated soft tissue injuries, the type of definitive surgical fixation and resources.

In most DRFs where surgery is indicated, a closed reduction and cast immobilization is recommended until the patient undergoes surgery. There are many different surgical approaches for DRF treatment. Implants include external fixators, palmar and/or dorsal plates, spanning plates and additional k-wires.

**Table 1:** Indications for surgical treatment and specific approaches [75]

General indications:	Indications for palmar plating:	Indications for dorsal plating:	Indications for external fixator:
• Palmar/dorsal shearing fractures	• Palmar shearing fracture	• Dorsal shearing fracture	• Temporary fixation in high-energy trauma
• Postreduction displacement	•Palmar comminuted fracture	• Dorsal radiocarpal dislocation type AO B2. 1–3	• Polytrauma
• Compression fractures of articular surface		• Centrally compressed (impacted) fragments	• Additional neutralizing device when stable fixation cannot be achieved with plating or wires
• Open fractures		• Displaced dorsal lunate facet fragment that cannot be reduced percutaneously	• Severe, open, contaminated fractures
• Associated compartment syndrome		• Associated complete carpal intrinsic ligamentous rupture	• Intraoperative distractor
• Associated neurovascular injury			
• Associated tendon injury			
• Dorsal bending fractures in high demand patients with postreduction displacement:	• 3 mm radial shortening • > 10° dorsal tilt • 2 mm articular incongruency		

### Palmar plating

Improved understanding of wrist biomechanics and the introduction of locking plates in the late ‘90s, have resulted in plate systems that facilitate fixation of the metaphyseal bone and also.

support specific columns with arrangements for the radial and intermediate columns.

Palmar implants are well covered by the pronator quadratus muscle. The watershed line represents the margin between the structures which are elevated proximally and the palmar wrist extrinsic ligaments. They should not be detached from the radius (to expose the joint surface) as this may destabilize the wrist. The placement of the locking plate on the palmar surface reduces plate related soft-tissue problems and the stability of these devices makes bone grafting of the metaphyseal bone defect unnecessary. Palmar plating with a locking implant allows anatomical restoration of length and rotation and secondary loss of reduction is less common [76]. In addition, early mobilization is possible with rapid return to function and less risk of complex regional pain syndrome.

### Dorsal plating

Indications for a dorsal approach are depicted in Table 1. The use of low-profile locking plates and screw heads on the dorsal side of the wrist has reduced soft-tissue irritation at this site. The approach between the extensor tendon compartments depends on the fracture pattern and must be carefully planned after assessing the radiographs and CT. Plate position can be orthogonal or parallel, depending on the articular displacement of the scaphoid facet [7, 76]. A dorsal arthrotomy can be performed parallel to the dorsal radial rim or between the larger articular fragments to inspect the articular surface and to look for any associated carpal injury. With contouring of the distal part of the plate, a precise fragment reduction can be achieved.

### External fixator and/or K-wires

Historically, external fixation was the treatment of choice using ligamentotaxis and distraction to reduce the fracture [77]. Nowadays, due to complications related to external fixation, this technique is mostly reserved for damage control principles.

TReduction is achieved with external fixators, K-wires, or a combination of both. The external fixator is usually applied as a joint bridging fixator but when the distal fragments are sufficiently large, it can be applied without bridging the wrist joint.

When an external fixator is used for definitive treatment, it is left in place until fracture healing is adequate so that redisplacement is unlikely, which is usually 6 weeks following surgery.

### Wrist spanning plate

In cases of extreme comminution, when it is not possible to anatomically reconstruct the joint, a temporary joint spanning plate across the radiocarpal joint surface may be considered. The proximal carpal row can be used as a template against which the fragments are reduced. It is important to visualize and retract extensors where they exit dorsal compartments, especially the EPL tendon, since risk of iatrogenic injury is present. Largely displaced fragments of the lunate facet and sigmoid notch should be acceptably reduced. The aim is to hold the multiple fragments in an acceptable alignment and relationship to the carpus and distal ulna until healing is achieved.The plate is removed once radiological fracture healing is confirmed, typically between 3 and 4 months. Some stiffness is expected, but functional outcomes are often surprisingly good [78, 79].

## Associated soft tissue injuries

Soft tissue injuries on the ipsilateral wrist are often associated with DRFs. Most common are distal radioulnar joint instability (DRUJ) and scapholunate (SL) ligament injuries. Radiocarpal fracture-dislocations are less frequent, but represent a very severe injury.

### Distal radioulnar joint instability

Following DRFs, DRUJ instability has been reported with widely varying incidences [80–82]. Although the optimal treatment method of acute DRUJ instability with DRFs is controversial, many studies suggest that DRUJ instability is a poor prognostic factor, often resulting in chronic pain, decreased ROM and decreased grip strength if underdiagnosed or left untreated [80]. DRUJ stability is mandatory for proper force transmission between the forearm and the wrist.

The distal radioulnar joint (DRUJ) is a complex anatomical structure with little bony stability of the sigmoid notch and is mostly stabilized with the dorsal and volar radioulnar ligaments (RUL) comprising the triangulofibrocartilage complex (TFCC). In addition, the distal oblique bundle of the interosseous membrane and the dorsal capsular ligaments contribute to stability. Predictive factors for DRUJ instability are reported to be coronal plane displacement, comminuted fracture patterns with palmar/dorsal avulsion fractures of the RUL, fractures involving the sigmoid notch and concomitant base fractures of the styloid process of the ulna [83]. A fracture of the tip of the styloid process is most often not associated with TFCC injury and subsequent instability of the DRUJ [83].

DRUJ instability following DRFs can be treated with an above-elbow cast or sugar-tongue cast in supination for 4–6 weeks, transfixation of the DRUJ with K-wire fixation or direct (arthroscopic) TFCC repair. Fixation of the ulnar styloid process should only be considered if there is a dislocated oblique base fracture which involves the fovea or a fracture involving the sigmoid notch with DRUJ instability. A recently published systematic review concludes that direct TFCC repair failed to show any superior clinical benefit with regards to ROM, grip strength or PROMs when compared to K-wire fixation or conservative treatment [80]. However, transfixation can result in malreduction with subsequent unfavourable outcomes and care should be taken when this is performed. Residual DRUJ instability after untreated TFCC injury following DRF fixation has been commonly reported [84]. Conflicting results are presented with regards to gripstrength, ROM and PROMs [80, 84, 85]. Clear recommendations are difficult to formulate; an above elbow-cast in supination might be the best solution with the evidence at present.

### Scapholunate ligament injury

Scapholunate (SL) ligament injury is reported to have an incidence up to 63% in patients with DRFs [86, 87]. The SL acts as a stabilizer between scaphoid and lunate bones and injury can results in scapholunate advanced collaps (SLAC) with subsequent radiocarpal arthritis if left untreated. This can lead to significant pain, recuded ROM, grip strength and PROMs, resulting in adebilitation condition for patients [88]. The degree of instability of the SL varies between dynamic and static instability. Clinically differentiating between these can be difficult in the acute setting. However, following DRF osteosynthesis with fluoroscopy in the operating room, static instability can be diagnosed, when the lunate does not follow the scaphoid in radioulnar deviation.

Treatment options are conservative with cast immobilization, K-wire fixation or direct (arthroscopic) repair with bone anchors or capsulodesis augmentation. A recent systematic review reports better SL angles and gaps following surgical treatment. In addition, significantly lower ROM in flexion in the surgical group is reported (53° versus 59°) and extension (59° versus 62°) were measured. PROMs were significantly better in the surgically treated group with DASH scores 9 versus 12 [87, 89]. Follow-up of the included studies is varying. The surgical repair is performed to gain function at short term, but moreover to prevent osteoarthritic changes in the future. Clear differences regarding the type of surgical treatment are not presented. The recommendation is therefore, that in DRFs with staticSL injury, this should be surgically addressed to warrant the best outcome for the near and far future.

### Radiocarpal (sub)luxation

Radiocarpal fracture-dislocations are uncommon with reported incidences of 0.2% of all DRFs [35, 90]. However, it is a complex injury with dislocation of the radiocarpal joint in dorsal or volar direction, resulting from high energy trauma. Two classifications are known; Dumontier et al. describe the presence of solely ligamentous injury (only a small cortical avulsion radius included) as type I and associated fractures as type II [91]. Moneim et al. differentiate type I as a radiocarpal fracture-dislocation without associated intercarpal dissociation, while type II has associated interparal dissociation [92]. This second type represents a more complex pattern and can be considered as a variation of a perilunate fracture-dislocation as described by Mayfield et al. [93]

In contrast to historical nonoperative treatment, nowadays, surgical treatment is thought to be mandatory to result in a stable, concentric and congruent wrist [94, 95]. All irreducible dislocations, open injuries and cases with neurovasculair impairment should acutely be treated surgically. In these cases, two-staged procedures with temporary external fixation should be considered, followed by scheduled secondary definitive reconstruction. Three principles are recommended for reconstruction:

1. Adequate reduction and fixation of the radiocarpal joint (including reduction and osteosynthesis of the DRF);
2. Identification and treatment of intercarpal injuries;
3. Stable repair of the osseous-ligamentous avulsions [96].

## Rehabilitation

Aftercare protocols differ significantly between countries and institutions. Furthermore, patient and fracture characteristics as well as surgeons specific preferences affect the applied aftercare protocols. Universal concepts of reduction, retention and rehabilitation apply both for non-operative and operative treated patients and a comprehensive rehabilitation protocol for DRF has been composed and provided in flowchart 1.

Before finishing cast immobilization period or before surgical treatment, no mobilization should be performed of the radiocarpal joint. Although, until removal of the cast, adjacent joints (shoulder, elbow and fingers) should be actively mobilized to retain strength and prevent stiffness [97].

After removal of the cast/fracture fixation, most patients are allowed to start with pain-adapted mobilization of the wrist in all planes. Initially, passive mobilization is allowed in a pain-free interval. In most operatively treated patients early passive mobilization of the wrist within the pain-free range is possible due to implant stability already within the first 10 days after intervention [98]. Earlier range of motion training does speed up functional recovery, however, long term outcome is not affected [99].

Therafter, patients can be cleared for active movement and increased loading Loading starts with light functional activities and progresses gradually. In general, after 6 weeks, partial load transitions to full load if radiological healing is confirmed. In the strengthening phase (after 10–12 weeks), targeted grip, forearm, and proprioceptive training is introduced. Sport without high load or impact is allowed after 12 weeks, with return to full contact or high-impact sport only after 3–4 months, depending on healing and functional recovery.

**Flowchart 1:**
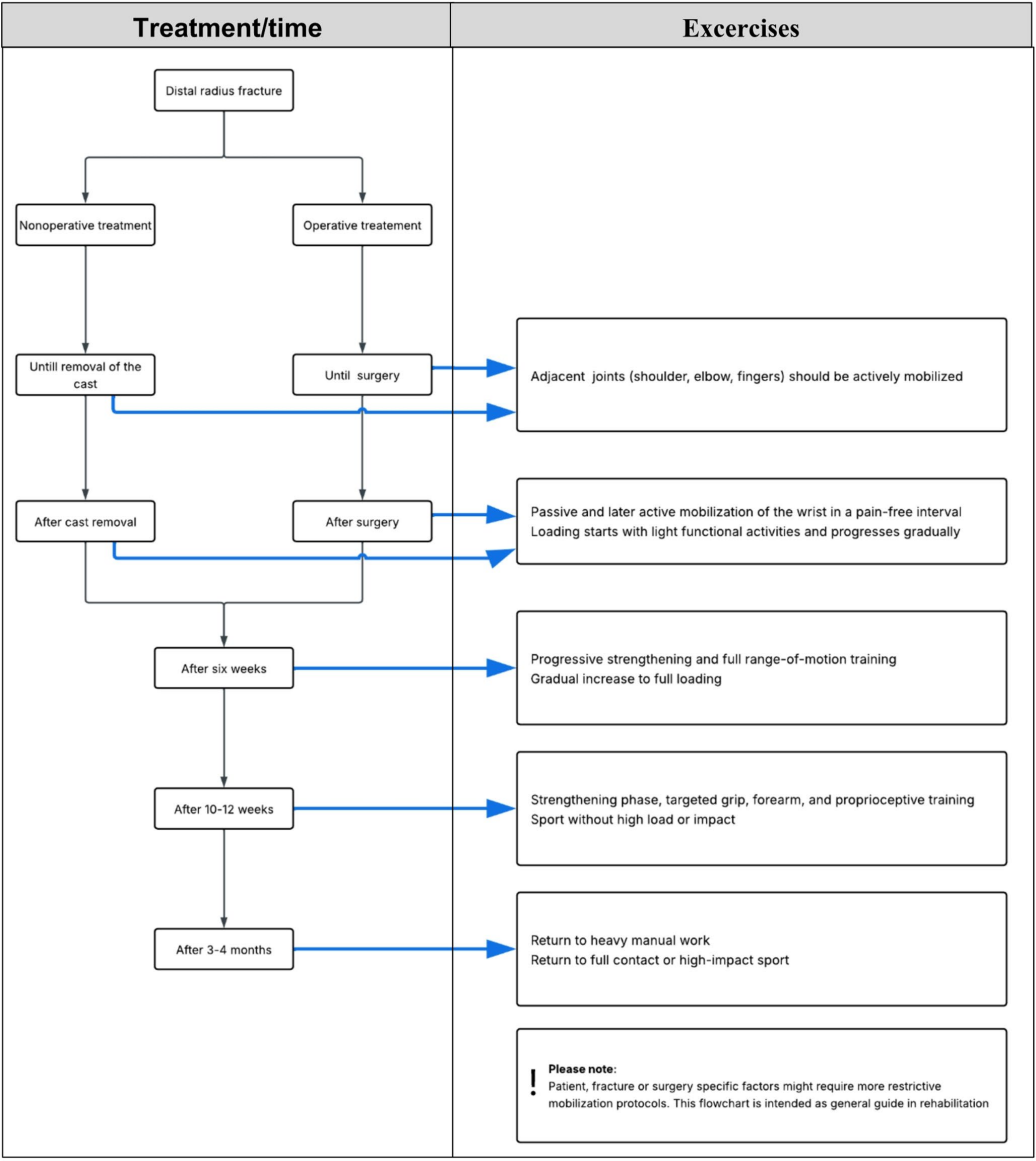
Rehabilitation protocol for non-operatively and operatively treated DRFs

Handtherapy and physical therapy specialists may be involved early after trauma to maintain functional capacities of adjacent joints including the fingers during the initial phase after surgery or in the case of non-operative management Supervised physical therapist is associated with better outcome than home care plans. Nevertheless, a large subgroup of patients, however, is not routinely referred to physical therapy during the immobilization phase [100].

Post-operative imaging is recommended after 6 weeks with thereafter intervals of 3 to 6 months depending on fracture complexity and joint involvement [101]. There is no evidence supporting more frequent post-operative imaging in extra-articular fractures, whereas in complex intra-articular fractures earlier imaging affect the aftercare protocol in a subset of patients [102]. And adequate intra-operative imaging may replace post-operative imaging in patients remaining asympotomatic.

### Outcome and patient expectations

It is important to inform the patients adequately about the expected aftercare protocol and functional recovery. A study from Quax et al. have demonstrated that complaints decrease from 60% after 6 weeks to 15% after 1 year [103]. Recovery is most profound within the first three months after DRFs, although improvement can occur even after one year [104–106]. Furthermore, there is a mismatch between objective decreased ROM after treatment and upper limb disability suggested by Aparicio et al. [107] The impact of immobilization of the wrist joint on adjacent joints, related muscles and compensatory mechanisms may partly explain this discrepancy [108].

## Implant removal

Indications for hardware removal differ between institutions and countries and evidence based guidelines are lacking. Common indications for hardwareremoval are flexor tendon injuries or tendinopathy, persistent pain, articular damage, infection, fracture healing issues and hardware irritation [109–111].

Flexor tendon damage is a frequent indication for plate removal and repair. In order to prevent flexor tendon irritation and injuries the Soong Classification system can be utilized to predict FPL irritation. This classification determines the height of the distal rim of the plate in concordance to the watershed line. With a Soong II or III, plate removal is recommended when union is achieved [112].

Controversy exists regarding indications for hardware removal in asymptomatic patients [113]. In a study on 806 patients with distal radius fractures, 252 underwent hardware removal surgery, of whom 73,8% was asymptomatic. Upon hardware removal, minor complications occurred in 10% of cases. Higher complication rates, however, have been described for elective implant removal of forearm plates [114]. Hence, it seems reasonable to be more reluctant when considering the removal of hardware.

According to a questionnaire study, patient satisfaction increased after hardware removal, and 93% of patients would opt for implant removal again, although in addition to these subjective findings no evidence supports the routine removal of hardware in asymptomatic patients without risk factors for flexor tendon issues [115]. More studies are required to determine which specific patient groups benefit from hardware removal after distal radius fracture fixation.

## Considerations in the elderly patient

In individuals aged 65 years and older, DRFs account for approximately 18% of all fractures [116, 117]. These injuries predominantly result from low-energy mechanisms such as falls from standing height, and their incidence is expected to rise concomitantly with global demographic shifts toward an aging population with associated increasing incidence of osteoporis [86, 117–120]. This trend poses increasing challenges to manage these fractures effectively, balancing functional outcomes, patient expectations, and healthcare resource utilization. This section aims to synthesize current evidence on treatment modalities for DRFs in elderly patients, providing practical recommendations in patient-centered and evidence-based care.

Traditionally, nonoperative management, primarily immobilization with casting, has been the standard treatment for DRFs in older adults [121, 122]. However, surgical intervention rates have increased substantially over the past two decades [123, 124]. Consequently, treatment decisions have to involve shared decision-making between surgeons and patients, considering individual functional goals [125].

In elderly, clinical decisions are influenced not only by fracture morphology but even more by patient-specific factors such as physiological age, activity level, comorbidities, and treatment preferences [114, 126]. It is important to emphasize that published studies used a different age to determ ‘elderly’. In many studies patients over 60 years of age were considered elderly. Nowadays however, patients in their sixth decade still fully participate in working life, sports and active recreation. In general, in patients of these elderly ages with a distal radius fractures, diagnosis and treatment of osteoprosis is recommended.

### Nonoperative treatment in the elderly

Nonoperative treatment involving casting remains appropriate for stable, non-displaced fractures. In the low demand elderly population, conservative treatment is also a good treatment option for (reduced) displaced fractures [5, 127]. This approach avoids surgical risks and is associated with acceptable functional outcomes [128–130]. The dilemma in the treatment of elderly patients with a DRF however, lies in whether or not to operate severely displaced fractures after insufficient reduction.

Several studies have highlighted the concerns of the treatment of elderly patients with a DRF. Two studies noted that while surgical treatment may offer improved anatomical outcomes, functional results do not always significantly surpass those of nonoperative treatment [121, 131]. Another demonstrated that cast immobilization was non-inferior to volar plating in patients aged 65 and older in terms of functional recovery. Evidence comparing operative and nonoperative management in this population shows mixed results, with some studies reporting comparable long-term outcomes but others suggesting improved grip strength and radiographic alignment following surgery [132, 133].

### Surgical treatment in the elderly

Surgical management of distal radius fractures in elderly patients presents distinct challenges that must be carefully considered during treatment planning. One of the primary concerns is poor bone quality due to osteoporosis, which compromises fixation stability and increases the risk of hardware failure or secondary displacement. Elderly patients also frequently present with multiple comorbidities such as cardiovascular disease, diabetes, and renal insufficiency, which elevate the risk of perioperative complications. Healing may also be delayed in this population due to age-related changes in bone metabolism and immune function. Additionally, frailty and limited mobility may hinder postoperative rehabilitation, delaying the return to independence.

When surgical treatment is preferred, specific considerations for elderly patients regarding the different types of fixation techniques should be taken into account:

- Palmar locking plate fixation is widely favored for intra-articular and unstable fractures because it provides rigid fixation, restores articular congruity, and enables early mobilization [134]. Also, volar plate fixation is preferred when early functional recovery is paramount [135].
- K-wire fixation have been described in the elderly for extra-articular or mildly displaced intra-articular fracture with shorter operative time (median ~ 23 min), and low infection rates [136]. Early postoperative recovery may be slower than with other methods due to delayed mobilization, impacting short-term grip strength and wrist motion; however, functional outcomes at 6 to 12 months are comparable to volar plating when rehabilitation is optimized [137, 138]. However, with low bone quality, we advise against K-wire fixation.
- External fixation might be used for comminuted fractures or when soft tissue conditions contraindicate internal fixation [139, 140]. It is characterized by reduced operative time. However, the risk of superficial infections and pin-site complications is higher, and early mobility may be limited.
- The wrist spanning plate can be a good alternative for external fixation, as described above. The aim is to hold the multiple fragments in an acceptable alignment and relationship to the carpus and distal ulna until healing is achieved.The plate is removed once radiological fracture healing is confirmed, typically between 3 and 4 months. Some stiffness is expected, but functional outcomes are often surprisingly good [78, 79].
- For select cases involving irreparable, highly comminuted intra-articular fractures, particularly in osteoporotic bone, wrist hemiarthroplasty has emerged as a promising alternative [141]. Early reports indicate low pain scores and preserved forearm rotation [142]. However, evidence remains limited, and further prospective studies are necessary to establish long-term outcomes and precise indications.

### Follow-up in the elderly

Functional outcome is not necessarily related to the quality of reduction, therefore follow up X-rays should only be performed if a clinical consequence can be derived [143]. In older patients, physical therapy is focused on general condition and return to self-sufficiency besides wrist specific rehabilitation. In addition, therapy focused on the shoulder and scapula may be beneficial to optimize outcome [144]. Different treatment options of the elderly patients with a DRFs is summarized in flowchart 2.

**Flowchart 2:**
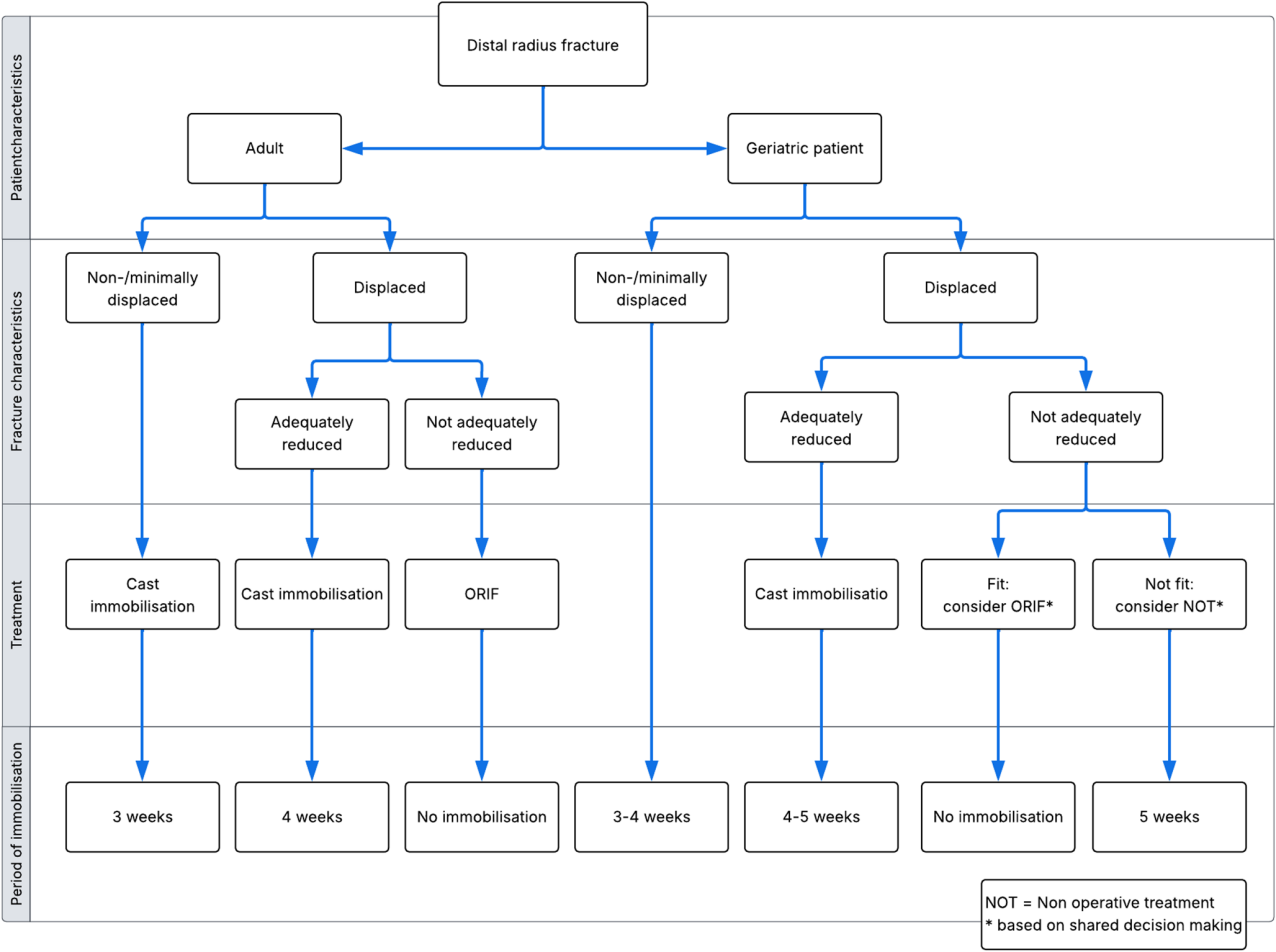
Algorithm for the treatment distal radius fractures

## Complications

The complication rate of DRFs described in literature ranges between 1 and 15% and depends on fracture characteristics and treatment [145–147].

### Acute complications

Although only 1% of DRFs comprise of open fractures, all complex DRFs are associated with soft-tissue injury [148]. To prevent further damage to the soft tissues, severely displaced fractures should be realigned as soon as possible. Gustilo grades 2 and 3 open fractures require prompt surgical management, with wash out and (temporary) stabilization [149]. Gustilo grade 1 open fractures, can be managed with non-surgical management and cast immobilization or delayed surgical treatment, based on the fracture characteristics.

Compartment syndrome of the forearm is a surgical emergency that is associated with high-energy trauma. Although this complication is very rare, with only case reports presented in literature, it requires prompt recognition and direct management by fasciotomy of all compartments of the forearm and release of the carpal tunnel [150].

Acute carpal tunnel syndrome is caused by an increase in pressure in the carpal tunnel, due to wrist deformity, displaced volar fragments, hematoma, edema or immobilization in extreme flexion or extension [151]. Complaints may start immediately after injury or sub-acutely with incidences reported of 3-8.6%. Complaints in the acute phase are often paresthesia of the fingers in the median nerve distribution, ranging from mild to complete absence of sensibility and motor function [152–154]. In case complaints resolve completely after reduction of the fracture at the emergency department, prompt surgical treatment is not necessary. In case of persistent complaints acute carpal tunnel release in combination with (temporary or definitive) fracture reduction is mandatory. The current literature does not provice a clear advice on the specific time frame in which release has to be performed.

### Short term complications

Some complications of DRFs are associated solely to operative treatment. Infection is a complication after plate fixation, which occurs in 3.1–8.3%. Standard treatment for fracture related infections (FRI) should be carried out with wash-out, cultures and antibiotic treatment in early FRIs [152, 153].

Radial, median and ulnar cutaneous nerves can be damaged during percutaneous or open surgical approaches. At risk is the cutaneous branch of radial nerve at the level of the radial styloid, where incisions might not be large enough to visualize and protect the nerve. The cutaneous branch of the median nerve should be protected during palmar approach by retracting all flexors together with the FCR tendon to the ulnar side. The incidence of damage to cutaneous nerves described in literature is rare (< 1%) [155]. However, one can imagine that this minor, yet impactful complication is being underreported.

Pin-site infection occurs in 7–8% [156, 157]. Tensioning of the skin around percutaneous K-wires or external fixators might be causing this or insufficient pin site care. Mostly pin track infections do not have severe clinical impact as the infection may be resolved after removal of the K-wires in the vast majority of the cases [158].

Articular screw penetration can occur in case of too distal implant positioning, inadequate approach and fragment visualization, or lack of proper imaging particularly in the tilted lateral view. If not recognized, penetration of screws can cause significant long-term complaints or increase in arthrosis. Screw penetration in DRUJ is even associated with an increase in QuickDASH/PRWE scores [159].

Tendon injuries after DRFs have been reported to occur in 1-6.4% [152, 153]. Extensor tendons can be irritated or ruptured due to dorsal screw penetration after volar plating [160]. Dorsally placed implants have little soft tissue cover and may irritate the overlying extensor tendons. However, low profile anatomically shaped implants recude this risk. Tendon rupture of flexors can be a risk, when palmar plates are placed distal to the watershed line, with the accompanying Soong classification warranting this [112]. (Late) ruptures of the EPL after non-operative treatment can occur due to the injury with contusion and subsequent ischemia of the tendon [161]. Although of severe impact when misdiagnosed, excellent clinical outcomes on DASH-score after tendon repair tendon ruptures after DRFs are reported [162].

Complex regional pain syndrome (CRPS) is characterized by autonomic nerve dysfunction, trophic changes, and functional impairment. The exact etiology is unknown. Incidences described in literature ranges mostly between 2 and 9%, however incidences up to 20% have been described [152, 163–165]. Research indicates that older age, female gender, complex fractures and those undergoing internal fixation are associated with an increased risk of CRPS. The occurrence of CRPS after a DRFs is significantly associated with diminished DASH-scores and increased VAS-scores [166].

Chronic carpal tunnel syndrome can occur sub-acute (1–12 weeks post-trauma) or late (> 12 weeks after trauma) and incidences up to 17% have been described. A six-item diagnostic algorithm helps to diagnose chronic CTS: median nerve paresthesia, atrophy of thenar muscles, reduced 2-point discrimination and positive provocation tests (Tinel’s test/Phalen’s test). Unlike acute CTS, in case of chronic CTS a conservative management can be started. If fracture displacement might be the case of the CTS, a correction osteotomy together with CTS release can be considered [154].

### Long term complications

Some degree of stiffness following a DRFs is common with all treatment modalities. Forceful distraction with an external fixator results in severe stiffness. Highly comminuted distal radius fractures form a risk factor, with a higher risk of malunion.REF.

Malunion of DRFs is described in up to 40% of non-operatively treated and 11% operatively treated DRFs [71, 167]. However, radiological malunion does not always correlate with clinical problems and in elderly patients with osteoporotic fractures it can be well tolerated [168]. Complaints following malunion entail diminished range of motion and/or DRUJ instability. Eventually progressive posttraumatic arthritis may lead to pain and diminished function. A corrective osteotomy should be considered to diminish or prevent these complaints, with more literature underlining the benefits of using 3D planning to optimize technical and functional outcomes [169–171].

Posttraumatic arthritis might be a long-term complication of DRF and is described in 32–65% of the patients [172–174]. Clinical outcome might be decreased in patients with posttraumatic arthrosis [168, 175]. Although one might expect an association to decreased PROMs as well, there is limited evidence on this subject [174].

## Summary 

Distal radius fractures comprise of 15% of all fractures in adults. Diagnostic evaluation consists of a medical history, clinical examination and radiographic imaging. If standard AP and lateral do not allow for adequate assessment of fracture displacement or intraarticular involvement, an additional CT-scan is indicated. Different classification systems are being used, of which the AO/ASIF is most anatomically detailed, the Fernandez classification focusses on mechanism of injury and displacement direction, while the four corner concept points out the ‘key-fragment’ with following the lunate. A combination of these classification systems can help describe the fracture pattern, but also help determine approach when surgical treatment is mandated.

Nonoperative treatment is advocated in non- or minimally displaced fractures. Reduction should be performed when a dislocated DRF is present. Criteria for acceptable reduction should be met (Fig. 1), when deciding on conservative treatment for DRFs. In young patients however, optimal anatomic reduction is preferred, and risk factors for secondary displacement should be taken into account. For non- or minimally displaced DRFs, 3 weeks of cast immobilisation is safe. For reduced DRFs, 4 weeks of cast immobilisation is safe. Follow-up radiographs at 7–14 days following reduction are advised.

Surgical treatment is indicated when dislocation exceeds radiographic parameters (Fig. 1). As stated before, these considerations may be different for elderly patients. Palmar and/or dorsal plating is the golden standard, based on the fracture pattern. External fixators with additional K-wires or spanning plates are used in highly comminuted DRFs and soft tissue problems. Especially, external fixators can be a bridge to definitive surgery, for example in polytrauma patients. The different algorithms for nonoperative and operative treatment are summarized in Flowchart 2.

Several associated soft tissue injuries are associated with DRFs. Suspicion of DRUJ instability should be present when highly comminuted DRFs, fractures involving the sigmoid notch, radio-ulnar ligament insertion or base fractures of the ulnar styloid are present. Following anatomical reduction and fixation of the DRF, DRUJ stability should be tested and treated with an above the elbow cast in supination, transfixation or direct refixation of the TFCC. SL injury presents with widening of the SL joint > 2 mm, testing under fluoroscopy to differentiate between static and dynamic injury can be performed. Only in static SL dissociations, surgical treatment should be considered. Radiocarpal luxation is uncommon, but should be addressed with adequate reduction and fixation of the radiocarpal joint, treatment of any intercarpal injuries and a stable repair of the osseous-ligamentous avulsions.

Rehabilitation protocol following DRFs are similar following nonoperative treatment after removal of the cast and surgical treatment following surgery. Gradual active mobilisation is advocated for the first 6 weeks, after which progressive strengthening and full range-of-motion training begins, which will be increased to maximum function (Flowchart 1). Guidement by physical therapy or handtherapy is advised. Total rehabilitation can take up to a year.

Implant removal should be performed when persistent pain, diminished range of motion due to hardware, tendon irritation, risk of tendon rupture, hardware irritation or infection are present. Controversy exists regarding routine hardware removal.

Considerations in the elderly patient with DRFs are based on the demand of the elderly patient. In general, diagnosis and treatment of osteoporosis is recommended (Flowchart 1). Nonoperative treatment is adequate for non- and minimally displaced, but also in displaced fractures for low demand patients with acceptable outcomes. In case of a fit elderly patient with a dislocated fracture, surgical treatment should be determined based on shared decision making with the patient. Poor bone quality with less purchase of osteosynthesis, higher complication rates, such as postoperative delirium, should be taken into account. Spanning plates or hemiarthroplasty are additional surgical options in the elderly.

Several complications following DRFs could present, ranging from acute compartment syndrome, carpal tunnel syndrome to short term complications following surgery such as infection, hardware related complications and nerve damage or tendon ruptures. Long term complications include stiffness, symptomatic malunion or posttraumatic arthritis. Adequate diagnosis of these complications is key to optimal treatment.

These recommendations describe up-to-date considerations regarding the diagnostic process, treatment, associated injuries and complications of DRFs.

## Data Availability

No datasets were generated or analysed during the current study.
